# Impact of efflorescence on internal salt precipitation dynamics during injection of gases in porous rocks

**DOI:** 10.1016/j.advwatres.2025.104984

**Published:** 2025-07

**Authors:** Gülce Kalyoncu Pakkaner, Veerle Cnudde, Hannelore Derluyn, Tom Bultreys

**Affiliations:** a PProGRess, Department of Geology, Ghent University, Ghent, Belgium; b UGCT, Department of Physics, Ghent University, Ghent, Belgium; c Department of Earth Sciences, Utrecht University, Utrecht, the Netherlands; d E2S UPPA, CNRS, LFCR, Universite de Pau et des Pays de l’Adour, Pau, France

**Keywords:** Salt precipitation, Microcomputed tomography, In situ flow experiment, Underground energy storage, Efflorescence

## Abstract

Porous geological formations play an important role as storage media for CO_2_ and H_2_. Brine in these formations can evaporate during gas injection, leading to salt precipitation. Estimating how and where salt precipitates is important to understand its influence on injectivity. So far, studies on gas injection scenarios primarily focused on formation dry-out inside the pore space. However, salt precipitation on external rock surfaces exposed to dry-out (e.g., the wellbore interface) can strongly influence drying dynamics as well. The occurrence of salt precipitation on these surfaces and its relation to the internal precipitation dynamics remain unclear. Therefore, we conducted experiments injecting dry gas into Bentheimer sandstone at two flow rates in the advective regime and for two different brine salinities. We tracked precipitation on both the gas injection surface and in the pores via *in-situ* μCT imaging. Salt precipitation on the surface occurred for all cases, and precipitation within the pores exhibited heterogeneous distribution, with higher salt accumulation at higher salinities and lower flow rates. Porosity decreased by less than 3 % for all cases, nevertheless accompanied by up to 20 % reduction in permeability. Our experiments suggest a strong correlation in time between precipitation dynamics within the pores and on the injection surface. We discovered a sudden decrease in brine concentration inside the pores during dry-out, with a simultaneous increase in the rate of precipitated salt volume on the surface, highlighting a previously unrecognized interaction. This work provides insight into complex drying and precipitation dynamics during gas injection, which carries important implications for well injectivity impairment in field operations.

## Highlights

• Salt precipitation in homogeneous sandstone during gas injection is quantified under shallow reservoir conditions.
• The significance of microporosity is noted, even in highly homogeneous sandstone.
• A strong correlation in time between the decrease in brine concentration in the pores and the rate of salt precipitation on the surface.
• The permeability of the salt crust has been estimated.

## 1 Introduction

As climate change is recognized to be one of the most serious global challenges, significant efforts are being made to mitigate it by reducing greenhouse gas emissions (IPCC, 2014). Porous geological formations such as saline aquifers and depleted reservoirs have a significant role in reducing greenhouse gas emissions. They provide storage space for CO_2_ to reduce its atmospheric levels (Tremosa et al., 2014; Bachu, 2015). Also, these porous formations can be used to store energy in different forms to balance out the supply and demand caused by the seasonal, fluctuating nature of renewable energy (Matos et al., 2019). Using porous geological formations either for CO_2_ sequestration or underground energy storage operations raises various complexities. Saline aquifers innately contain brine in the pore space. The equilibrium between the formation liquid and reservoir rock is altered when the gas is introduced into the pore space, potentially resulting in chemical and physical induced effects. Drying of brine in the porous medium is one of these effects, which can happen during gas injection. Due to the mass transfer between the formation water and the injected (dry) gas stream, the salt concentration in the brine increases and eventually reaches the solubility limit which leads to salt precipitation. The precipitated salt can block specific regions of the porous matrix of the reservoir, thereby reducing its storage capacity (Miri and Hellevang, 2016; Hajiabadi et al., 2021; Cui et al., 2023; Sun et al., 2024). Salt precipitation was reported as a substantial risk factor for injectivity impairment in gas production wells (Kleinitz et al., 2003; Giorgis et al., 2007), and CO_2_ sequestration in saline aquifers (Baumann et al., 2014; Grude et al., 2014; Smith et al., 2022).

Existing studies, particularly in the context of CO_2_ sequestration, have mainly focused on determining the location of the precipitation inside the pores and the overall effect of this precipitation on injectivity by explaining the physical mechanisms of salt precipitation during gas injection scenarios. Salt precipitation is driven by the balance between advection, evaporation, capillary fluid flow, and diffusion, which act at different time and length scales (Andre et al., 2014; Peysson et al., 2014). The interplay between these intricate physical mechanisms defines the macroscopic precipitation pattern in the pore space, and how the precipitated salt affects the injectivity accordingly (Andre et al., 2014; Ott et al., 2015; Miri and Hellevang, 2016). When the gas is first injected, it displaces the residing brine by viscous forces. After the liquid phase becomes stagnant, the evaporation flux becomes dominant, starting from the injection surface. This establishes a capillary pressure gradient between the wet region and the drying region in the pore space. Once this gradient overcomes the viscous pressure gradient, brine starts to flow back toward the injection surface (Giorgis et al., 2007; André et al., 2014; Roels et al., 2014; Miri and Hellevang, 2016). Capillary-driven backflow of brine can transport salt to the pores in the neighborhood of the injection surface, which can ultimately result in the precipitation of salt exceeding the amount estimated from the initial salinity (Giorgis et al., 2007; Pruess and Muller, 2009; Bacci et al., 2011; Ott et al., 2011, 2021; Norouzi et al., 2021; Zhang et al., 2024). The balance between evaporation, backflow and salt diffusion can lead to either homogeneous or heterogeneous precipitation (Giorgis et al., 2007; Peysson et al., 2014; Andre et al., 2014; Ott et al., 2014, 2015; Jeddizahed and Rostami, 2016) along the flow direction. Predicting this balance for specific pore structures, flow rates and thermodynamic conditions is however still challenging (Miri and Hellevang, 2016). This is particularly the case for flammable gases at reservoir pressure-temperature conditions, due to the experimental challenges this entails (Cui et al., 2016; Zhang et al., 2019; Tang et al., 2023; He et al., 2024a). Along with the interplay between different transport phenomena, precipitation dynamics are also affected by the properties of the porous media and by the type and initial concentration of salt (Shokri et al., 2024). The precipitated salt volume and its effect on permeability increase with the salinity of the formation fluid (Peysson et al., 2011; Andre et al., 2014; Jeddizahed and Rostami, 2016; Sokama-Neuyam et al., 2019; Md Yusof et al., 2021, 2022; He et al., 2022; Tang et al., 2023, 2024a; He et al., 2024b). The heterogeneity of the porous matrix also has an impact on the precipitated salt volume, as the transport of brine from low to high permeability regions increases the overall amount of salt precipitation (Ott et al., 2014; Ren et al., 2018; Akindipe et al., 2021).

Depending on the properties of the rock and the prevailing precipitation regime, salt clogging within the pores can lead to a significant reduction in absolute permeability, even when the change in porosity is relatively minor (Bacci et al., 2011; Peysson, 2012; Peysson et al., 2014; Roels et al., 2014; Edem et al., 2020). For example, studies with CH_4_ (Tang et al., 2015), CO_2_ (Bacci et al., 2013) and H_2_ (Bijay et al., 2024) have indicated porosity reductions in the range of 5–14 % can correspond with 75–83 % reductions in permeability. In addition to implications of the absolute permeability change due to precipitation, injectivity also depends on gas relative permeability (Liu et al., 2013). This expresses how strongly gas flow is inhibited by the presence of (remaining) brine in the pores and may hence increase during drying as the volume of brine in the pores decreases (Ott et al., 2012). Multiple studies showed that the increase in gas relative permeability can counterbalance the decrease in the absolute permeability caused by salt precipitation, yielding no injectivity impairment (Ott et al., 2011; Ott et al., 2012; Roels et al., 2014). Increases in gas relative permeability were linked to the gas flow paths remaining open due to preferentially precipitation in pores originally occupied by brine (Ott et al., 2011; Ott et al., 2012; Roels et al., 2014). This counteracting effect of relative permeability on permeability reduction was not observed in other studies (Wang et al., 2009, 2010). Hence, predictions of how injectivity is affected by salt precipitation in the pore space remain highly uncertain due to the complexity of both absolute and relative permeability effects.

The existing literature on the evaluation of dry-out during gas injection into a porous matrix mainly focuses on precipitation inside the pores, i.e., subflorescence. A limited number of gas injection studies (on sandstone) have reported that salt precipitation also occurs on the injection surface, a phenomenon called efflorescence (Bacci et al., 2011; Peysson, 2012; Jeddizahed and Rostami, 2016; Sokama-Neuyam et al., 2019). While these studies identify the overall effect of precipitation on injectivity, they did not simultaneously monitor the internal dry-out mechanisms within the pores while salt precipitates on the surface. Consequently, the potential influence of efflorescence on internal precipitation remains unknown, highlighting a critical knowledge gap in gas injection operations, particularly in underground storage applications. In these settings, efflorescence could play an important role, as it may occur in the contact between the reservoir rock and the well, requiring costly down-hole maintenance. Moreover, it has been previously discussed that efflorescence induced by gas injection may significantly reduce permeability (Peysson, 2012). Although external salt precipitation was less focused on in gas injection scenarios, it is well known from experiments under diffusive drying conditions, i.e. where water vapor leaves the porous structure via diffusion instead of by advective gas flow in gas storage operations, that there is a complex interplay between sub- and efflorescence. This has been investigated in other fields, including the weathering of building stones (Desarnaud et al., 2015; Lubelli et al., 2018; Derluyn et al., 2019) and soil degradation (Daliakopoulos et al., 2016; Dai et al., 2022).

Diffusive drying consists of a first stage dominated by capillary-driven brine transport to the external surface where water evaporates from it, followed by a second phase characterized by vapor diffusion through the pore space (Guglielmini et al., 2008; Shokri et al., 2024). Both diffusive and gas-injection drying can thus lead to (capillary) transport of salt towards a sample’s external surface, potentially causing efflorescence (Peysson, 2012). However, the drying rate for gas-injection depends strongly on the gas injection rate, hence establishing a different balance between evaporation, capillary transport and salt diffusion than under diffusive drying (Peysson et al., 2011). Furthermore, the injection rate also affects the extent of the dry-out zone (i.e. the zone before the gas becomes saturated with vapor), and hence the inhomogeneity of the drying rate along the flow direction. These considerations may significantly impact the resulting precipitation patterns that occur in both types of drying (Huinink et al., 2002; Peysson et al., 2014; Desarnaud et al., 2015).

In the studies concerning diffusive drying, the occurrence of subflorescence or efflorescence is linked to several factors, including the salt type, the porosity of the medium, and the prevailing environmental conditions (Scherer, 2004; Espinosa-Marzal and Scherer, 2008; Brito and Diaz Gonçalves, 2013). Depending on the pore size, two distinct patterns of efflorescence—characterized as crusty and patchy—exhibit varying effects on the evaporation rate (Eloukabi et al., 2013). Crusty efflorescence can completely cover the evaporation surface and eventually block evaporation, whereas patchy efflorescence exists in discrete locations on the surface, causing minimal impact on the evaporation rate (Nachshon et al., 2011; Veran-Tissoires and Prat, 2014). Interestingly, efflorescence can even enhance the evaporation rate by drawing water from deeper within the porous medium (Sghaier and Prat, 2009; Eloukabi et al., 2011; Veran-Tissoires et al., 2012). However, under advection-dominated conditions of gas injection, the dynamics of precipitation involving salt crystals formed on the surface remain unclear, along with their resultant effect on the pattern of salt precipitation in the pores.

This study investigates the link between efflorescence and the internal dry-out dynamics within the pore space for gas injection scenarios with a focus on subsurface storage operations. To this end, we conducted a series of gas injection experiments on brine-saturated mini cores of Bentheimer sandstone at 30 bar and 40 °C, simulating shallow storage conditions (Indro et al., 2024). The experiments were performed with nitrogen gas as an analog to different gases such as H_2_ and CO_2_, due to its more straightforward handling in the laboratory. While the impact of using this analog remains to be validated in further work, the physics of salt precipitation are generally expected to be similar for different gases, as long as dimensionless numbers are used to scale e.g. the ratio between advective and diffusive transport in function of the different thermodynamical gas properties (Miri and Hellevang, 2016). Moreover, N_2_ is one of the suggested cushion gases in underground hydrogen operations (Pfeiffer and Bauer, 2015; Shoushtari et al., 2023), which highlights the relevance of the findings of the study. Varying injection rates of N_2_ and initial brine concentrations were tested to explore the dependence of the efflorescence on these parameters. The injection rates were chosen in a regime where heterogeneous precipitation within the pores and salt precipitation on the surface were expected. During gas injection, the sample in the flow cell was monitored via *in-situ* micro-CT imaging, covering the injection surface and the pores in a region of approximately 6 mm in height. This way, the temporal and spatial evolutions of the gas, brine, and salt distribution were obtained for different states of dry-out. The results of this study aim to clarify the relation between efflorescence and internal dry-out mechanisms that are active inside the pores in gas injection scenarios.

## 2 Materials and methods

### 2.1 Rock samples and fluids

As a proxy sample for reservoir sandstones, Bentheimer sandstone was used. This well-sorted German sandstone exhibits a high quartz content, featuring a homogeneous pore structure with a porosity of 0.20–0.26 and permeability of 5.13 × 10^–13^ m^2^ – 2.98 × 10^–12^ m^2^ (Peksa et al., 2015). Three cylindrical microcores were drilled from the same block and left to dry in an oven at 40 ^o^ C for 24 h (a picture of the three samples is available in the Supplementary Materials, Fig. S1). The dimensions and water permeabilities of the samples were measured prior to the experiments and can be found in Table 1.

**Table 1:** Physical and intrinsic properties of samples.

Property	Sample 1	Sample 2	Sample 3
Diameter (mm)	6	6	6
Length (mm)	25	25	23
Porosity (-)	0.22	0.20	0.19
Water permeability (m^2^ x 10^–12^)	1.45	1.46	1.44

The salt solution was prepared by dissolving potassium iodide (KI) salt (VWR Chemicals, USA) in deionized water. Although the main constituent of brine in saline aquifers is NaCl (Niemi et al., 2017), in this study, we used KI solution due to the high atomic number of iodine, providing a distinguishable X-ray contrast that depends on salt concentration (either dissolved or precipitated) in each voxel. KI crystallizes in a cubic structure, similar to sodium chloride’s halite structure (Thomas, 1997). Furthermore, the trend of saturated vapor pressure of aqueous solutions of KI and NaCl in the function of salt concentration was observed to be similar (Shokri-Kuehni et al., 2017a). Finally, another factor influencing the salt precipitation behavior is the solution viscosity. Literature data shows that, at the temperature used in our experiment, the viscosity of NaCl varies between 6.9 × 10^–4^ Pa.s and 7.4 × 10^–4^ Pa.s for relevant salt concentrations (Kestin and Shankland, 1984), compared to 7 × 10^–4^ Pa.s for KI (Goldsack and Franchetto, 1978). The impact of the difference in viscosity is thus deemed negligible.

During gas injection, dry N_2_ was employed. Since we used N_2_ as an analog to different gases relevant to underground storage applications, such as CO_2_, H_2_, and CH_4_, it is important to compare their physical properties that influence salt precipitation (Table 2). At the given temperature and pressure, the viscosity of N_2_ is comparable to that of CO_2_ and CH_4_ but an order of magnitude higher than H_2_. Additionally, H_2_ has a significantly lower density compared to N_2_. All gases exhibit similar interfacial tension with N_2_, except for CO_2_, which has a slightly lower interfacial tension with brine. The solubilities of N_2_, H_2_, and CH_4_ in water are similar but lower than that of CO_2_, making CO_2_ more prone to chemical interactions, such as carbonate precipitation. While these differences could impact the extent of salt precipitation and the rate of evaporation, the main findings of this study can be extended across different gases.

**Table 2:** Physical properties of relevant storage gases at given experimental conditions.

Gas	Density* (kg.m^-3^)	Viscosity* (Pa.s)	Brine Solubility⁎⁎ (mol. kg^-1^)	Interfacial Tension^a^^,^⁎⁎⁎ (N m^-1^)
H_2_	2.3	9.2 × 10^–6^	0.017	0.072
CO_2_	58.9	1.6 × 10^–5^	0.3	0.055
CH_4_	19.3	1.2 × 10^–5^	0.0028	0.064
N_2_	32.3	1.9 × 10^–5^	0.0095	0.07

a Average values for the brine concentration range used in the study.

⁎ National Institute of Standards and Technology, 2025.

⁎⁎ Bonto and Andreasen, 2024.

⁎⁎⁎ (H_2_: Hosseini et al., 2022; CO_2_: Bachu and Bennion, 2009; CH_4_: Kashefi et al., 2016; N_2_: Pan and Trusler, 2022).

### 2.2 Flow apparatus and experimental procedure

**Fig. 1:**
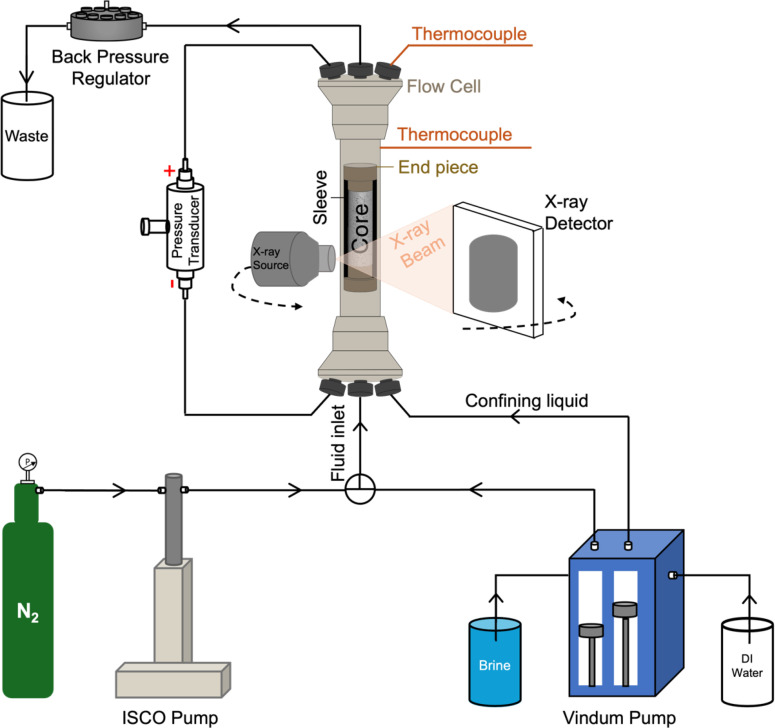
A schematic of the experimental setup, not drawn to scale.

A schematic illustration of the experimental configuration is given in Fig. 1. In this study, two different KI concentrations and flow rates were tested. The salt solution concentrations are selected within the range of real aquifer salinities (Saraji et al., 2014), while they remain below the solubility limit of KI in water at 40°C, which is 61 wt.% (Pawar et al., 2010). Flowrates were determined based on non-dimensional Peclet number (Pe) which shows the ratio of the advective to diffusive transport rates (Eloukabi et al., 2011; Peclet, 1827):

(1) $$Pe=\;\frac{uL}{D}$$

where *u* is the interstitial velocity of the injected gas (Darcy velocity/sample porosity, m/s), *L* is the characteristic length (m), and *D* is the diffusion coefficient (m^2^/s). The diffusion coefficient of KI in water was taken as 1.9 × 10^–9^ m^2^/s (Dunlop and Stokes, 1951).

Competition between advective and diffusive transport defines where salt predominantly precipitates. Studies conducted in drying of saline porous media under diffusive boundary conditions, i.e. natural convection, showed that if diffusion dominates over advection (Pe<1), salt can precipitate as subflorescence. The dominance of advection (Pe>1), on the other hand, yields salt precipitation as efflorescence (Guglielmini et al., 2008; Sghair and Prat, 2009; Veran-Tissoires et al., 2012; Shokri, 2014). Given that salt precipitation on the surface and its effects on the dry-out dynamics were the major interests of this study, the tested injection rates were both selected to satisfy Pe>1 conditions. The resulting Pe numbers were calculated as 3.8 and 38 for lower and higher injection rates, respectively.

Salt concentrations and flow rates used in each case are provided in Table 3. For each case, the same preliminary steps and procedures were followed as described below.

**Table 3:** Experimental parameters used for different cases.

Property	Experiment with Sample 1	Experiment with Sample 2	Experiment with Sample 3
N_2_ injection rate (ml/min)	0.8	0.8	0.08
Concentration of KI solution			
(% wt.)	20	10	20
(mol/L)	1.4	0.7	1.4

Each sample was placed in a Fluoroelastomer Viton sleeve and mounted into a Polyetheretherketon (PEEK) hassler-type X-ray transparent flow cell (RS Systems, Norway). The flow cell contained two inlet and outlet ports. One set of inlet and outlet ports was used for pressure measurement, while the other ports were used for fluid injection. The flow cell was positioned vertically with the bottom part functioning as the inlet, at the stage of the Environmental micro-CT (EMCT) scanner at Ghent University’s Centre for X-ray Tomography (UGCT; Dierick et al., 2014; Bultreys et al., 2016). This scanner features a rotating source and a detector gantry, which is advantageous to prevent unintended movements of the flow cell and the connected lines through it during image acquisition.

To ensure that the injected fluid penetrates the rock matrix without flowing along the sample boundaries, a confining pressure, which was always kept 15 bar above the pore pressure, was applied to the Viton sleeve around the sample. Before the start of the injection, the sample was fully saturated with brine, using a high-pressure syringe pump (VP12 high-pressure pump, Vindum Engineering, USA). For dry N_2_ injection, a high-pressure syringe pump (500D, Teledyne ISCO, USA) was used. A differential pressure transducer (Keller PD-33X, Switzerland) was connected to the sample inlet and outlet to record the pressure drop over the sample during gas injection. To maintain a constant outlet pressure at 30 bar, a low flow back pressure regulator (Equilibar, USA) was connected to the outlet of the flow cell. The sample was heated to a constant temperature of 40℃ using a Polyimide film-insulated flexible heater (Omega, UK) that was wrapped around the flow cell. Thermocouples- one inserted into the flow cell to measure the sample temperature and the other placed on the surface of the flow cell as the overheating control, were installed to regulate the system temperature using a PID temperature control unit (Genlab, UK). The experimental procedure is as follows:

1. The whole sample was scanned in a dry state.
2. The back pressure was increased to 30 bar. The sample’s absolute N_2_ permeability was measured by recording the pressure drop values across the sample at five flow rates varying between 1 ml/min and 10 ml/min.
3. The sample was depressurized and saturated with approximately 100 PV of deionized water. Then, the water permeability of the sample was measured at ambient pressure.
4. The sample was saturated with approximately 500 PV of brine at the designated concentration for each set, and a scan was conducted to confirm a full saturation state.
5. After completing brine saturation, the sample was heated in a controlled way up to 40℃.
6. Once the sample temperature became stable, the system was gradually pressurized back to 30 bars by initiating the N_2_ Injection.
7. During the continuous gas injection, to monitor the evolution of the dry-out process, the sample was scanned at specific time intervals of 2 to 4 h depending on the employed injection flow rate.
8. Upon the indication of complete dry-out of the sample, evidenced by no distinction in the differential images between two consecutive time points, the N_2_ permeability was measured following the procedure given in step 2.
9. The gas injection was stopped and the whole sample was scanned.

### 2.3 X-ray μ-CT imaging and image processing

For the dry and after-precipitation states, the entire length of the sample was scanned with a voxel size of 6.3^3^ µm³. The scans featured an exposure time of 1.1 s per radiograph for a total of 2400 projections per turn with 4 averages, repeated at 4 different vertical positions along the sample. The X-ray source accelerating voltage was 120 kV, and the target power was set at 8 W. For the scans of the brine-saturated state and during gas injection, images of only the bottom part of the sample were made (approximately 6 mm in height) to speed up the scanning. These were acquired with 2100 projections at 4 averages, keeping the other parameters constant.

The tomographic reconstruction of the raw image data was executed with the in-house developed software Octopus Reconstruction. Then, the non-local means filter (Buades et al., 2008) was applied to reduce the noise in the images (Avizo 2022.2, ThermoFisher, France). The image of the dry scan of the sample was segmented into pores and grains using Trainable Weka Segmentation (Schindelin et al., 2012; Arganda-Carreras et al., 2017). Images that were acquired at different timesteps of the experiment were registered and resampled to the dry-state reference images (Avizo 2022.2, ThermoFisher, France). Segmentation of the liquid and gas phases on the images acquired during injection was done with interactive thresholding, using the dry-state segmented image as a mask for the pore space. The sensitivity analysis for the segmentation of selected data points was done, and the resulting error bars were found to be statistically insignificant. Further information can be found in Supplementary Materials (Table S1). For the state after precipitation, salt in the sample was segmented by applying the same method (Avizo 2022.2). Note that high-concentration brine and crystallized salt are not straightforward to distinguish in the images.

Due to the high X-ray absorption coefficient of iodine, changes in the greyscale value of the brine phase can be linked to KI content. Therefore, mean greyscale values of the voxels that belong to the brine phase were used as a proxy for brine concentration. To do this, volume-averaged greyscale values were computed and normalized by the highest mean greyscale value obtained in the dynamic images collected over the injection, using Eqn 2 (Van Offenwert et al., 2019).

(2) $$proxy\;concentration=\;\frac{GV_{brine}-GV_{water}}{GV_{max}-GV_{water}}$$

Here, *GV_brine_* refers to the average greyscale value of brine within the pore space at a specific time point during the injection. *GV_water_* represents the average greyscale value of water within the pore space, obtained from the image of water saturated sample. *GV_max_* corresponds to the maximum average greyscale value of the analyzed pore space observed in the dynamic images collected throughout the injection. It should be noted that time point where maximum average greyscale value (*GV_max_*) was obtained differs for micro and macropores, which will be detailed in the following. Here, micropores refer to under-resolved pores, i.e. in patches of clay with a size smaller than the voxel size of the images (6 µm^3^). Macropores refer to the pores identified with the image resolution, with average and maximum pore size of 30 and 100 µm, respectively.

## 3 Results and discussions

### 3.1 Observations on the physical mechanisms of salt precipitation

To summarize the mechanisms occurring during dry-out, the outcome of the experiment that was conducted with the high N_2_ injection rate and the high concentration of KI solution (sample 1) is analyzed and discussed here first.

**Fig. 2:**
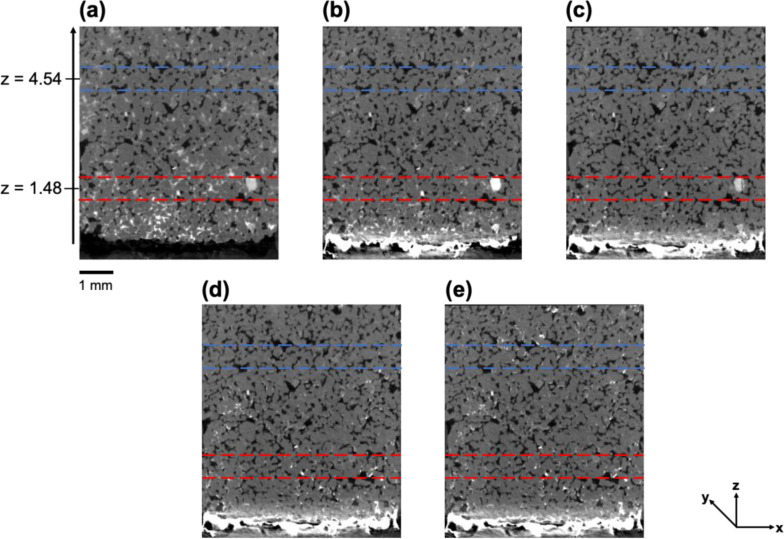
Evolution of the dry-out for sample 1, where N_2_ was injected at a high flow rate to the sample initially saturated with high-concentration brine. Bands with blue and red dashed lines represent regions away from and near the injection surface, respectively. *z* = 0 corresponds to the injection surface. Vertical slices illustrate changes at the following time steps: (a) *t* = 1.3 h, (b) *t* = 5.6 h, (c) *t* = 6.6 h, (d) *t* = 7.6 h, and (e) *t* = 9 h.

**Fig. 3:**
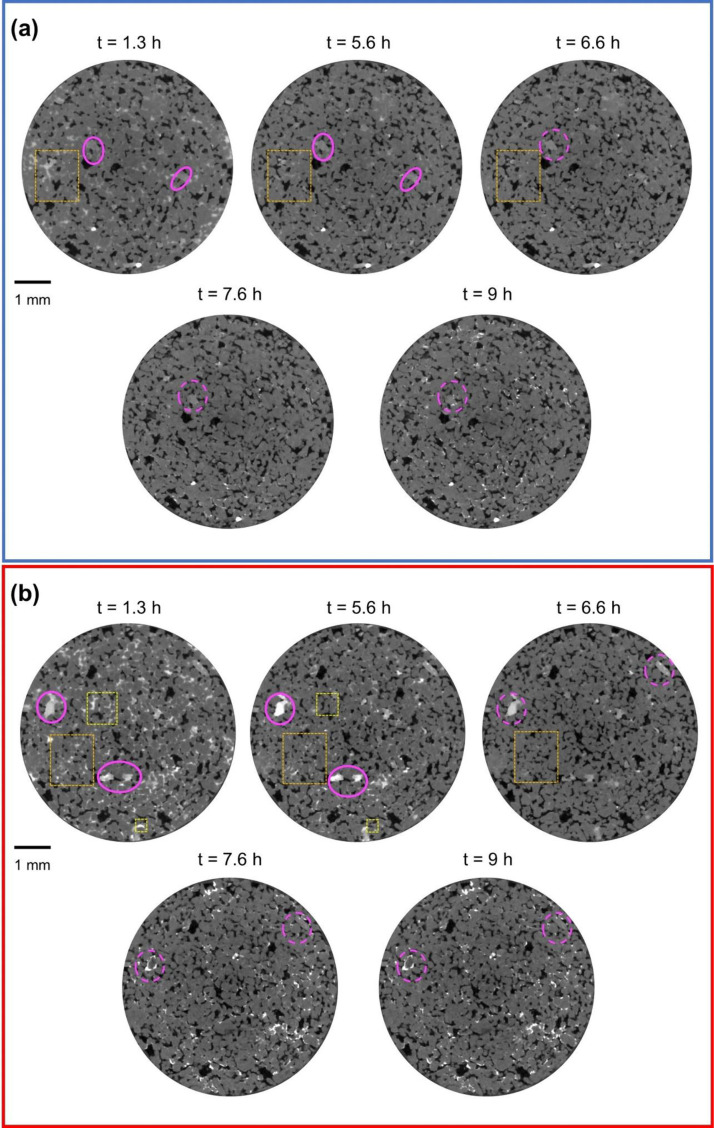
Horizontal slices showing the temporal evolution of dry-out. Slices correspond to the position of (a) *z* = 4.54 mm, and (b) *z* = 1.48 mm as the middle of blue and red bands given in Fig. 2, respectively.

**Fig. 4:**
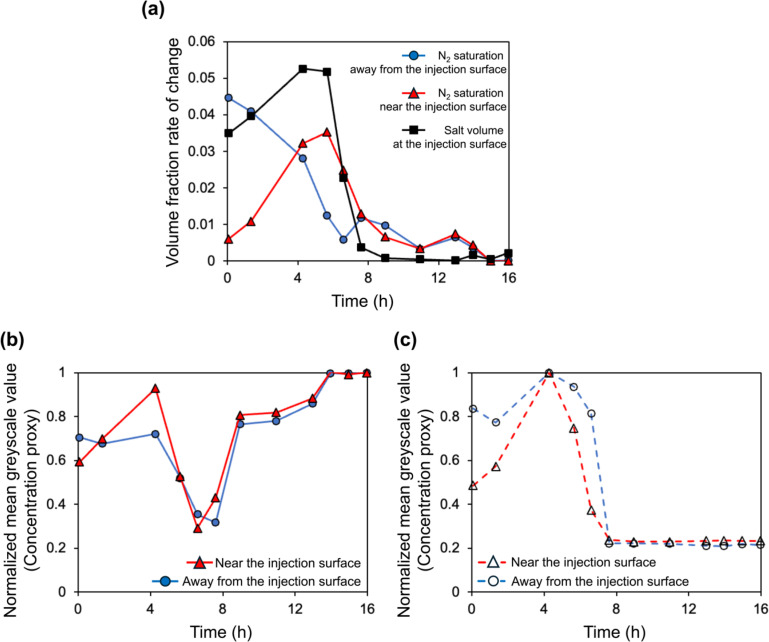
Quantitative interpretation of the summarized dynamics. (a) Rate of change of the average N_2_ saturation, together with the rate of change of the volume fraction of the salt precipitation on the surface. Normalized mean greyscale values of high-intensity phase as the concentration proxy for (b) macropores, and (c) micropores in the clay. Blue and red colors represent the average values calculated for the blue and red bands depicted in Fig. 2, respectively. The black color in (a) represents the external salt precipitation on the injection surface.

Upon the gas injection, the first physical mechanism that contributed to dry-out was the immiscible two-phase displacement governed by the viscous pressure gradient. This was monitored through the pressure readings provided by the differential pressure transducer (see Fig. S2 in Supplementary Materials). These readings first showed the breakthrough pressure and then exhibited a rapid decrease which signals the end of the primary drainage. This was taken as the initial time step (*t* = 0) of the evaporative drying stage. Image analysis showed that after primary drainage, brine was only left remaining in relatively smaller pores since larger pores were already invaded by gas due to having lower gas-entry pressure. From this point onward until the complete dry-out is reached, changes in N_2_ saturation, brine concentration, and salt precipitation in the pores and on the surface are given with vertical slices through the scans at different time steps in Fig. 2. To illustrate the spatial variations in the gas saturation and brine concentration across the field of view during drying more accurately, two bands were selected as representatives of changes at the pores near (zone enclosed by red-dotted lines) and away from (zone enclosed by blue-dotted lines) the injection surface, respectively. Cross-sections from the midpoints of red (*z* = 1.48 mm) and blue (*z* = 4.54 mm) bands for each time step are provided in Fig. 3 for a clearer visual representation. Observations are quantitatively represented for the red and blue bands in Fig. 4. Fig. 4-a shows the rate of change of the volume of salt precipitated on the injection surface, along with the volume fraction rate of change of N_2_ saturation in the pore space. We used Eqn 2 to show the variations in the greyscale values of macropores, and micropores, in terms of the proxy concentration in Fig. 4-b and 4-c, respectively. The main findings from these figures can be summarized as follows:

• After the primary drainage ended (*t* = 0), the evaporative flux caused more brine displacement in the pores far from the injection surface than near the injection surface (Fig. 4-a). At the following time steps, the brine concentration in the non-swept pores near the injection surface increased (Fig. 2-a). In the next stages of evaporative drying (from *t* = 1.3 h to 4.3 h) the increase in the concentration in macropores (Fig. 4-b) near the injection surface is smaller than in the micropores (Fig. 4-c)
• As the injection continued, salt precipitation on the injection surface became apparent (Fig. 2-b). Simultaneously, there was a displacement of high-concentration brine within the pores near the injection surface (dashed yellow boxes *t* = 1.3 h and *t* = 5.6 h in Fig. 3-b), which contributed to an accelerated increase of the N_2_ saturation in the pores (Fig. 4-a). Given that salt precipitation on the surface creates an additional porous structure (Shokri-Kuehni et al., 2017b), the high-concentration brine in the pores could potentially be moved upward to the injection surface where it contributes to crystal growth.
• After 5.6 h of N_2_ injection, the growth rate of the volume of precipitated salt on the surface increased substantially (Fig. 4-a). Concurrently, an unexpected decrease in brine concentration was observed. Indeed, the grayscale intensity of brine in macropores (Fig. 4-b) and micropores (Fig. 4-c) decreased significantly, even dipping below its value at the initial time step (Fig. 2-a). The decrease in grayscale intensity of the brine phase in pores away from (Fig. 3-a) and near (Fig. 3-b) the injection surface was also highlighted with the dashed orange boxes from *t* = 1.3 h to *t* = 6.6 h. Similar observations on concentration decrease in the highly concentrated brine within the pores at the later stages of dry-out were made in a recent study where gas injection through brine-saturated sandstone was conducted via in situ micro-CT imaging by Akindipe et al. (2021). This phenomenon was termed “reverse solute diffusion”, and it was explained as the transport of salt ions from low- to high-concentration brine via connected liquid films, driven by the high interfacial tension between the supersaturated brine and the injected gas at the evaporation front (Akindipe et al., 2021). Given the fact that the focus of their study was primarily on the dynamics of salt precipitation within the pores, no observation was provided regarding the injection surface. However, in our study, images were acquired for both the injection surface as well as the pores within the sample allowing us to observe the strong correlation in time between the decrease in brine concentration in the pores and the growth rate of the salt precipitation on the surface. Also, we observed that the brine concentration decreases in the pores near to injection surface as well (Fig. 4-b and c), whereas Akindipe et al. assumed that salt precipitation had already started there (Akindipe et al., 2021). Our observations lead to the following alternative hypothesis for the concentration decrease: the brine may become undersaturated due to a locally very fast precipitation rate on the surface, in particular, related to the time scale of diffusion over the length of the sample, causing a temporary “undershoot” in the salt concentration. Another possible explanation regarding the decreased brine concentration could be the exothermic nature of the crystallization of KI (Apelblat and Korin, 1998), which may locally disrupt the thermal equilibrium within the system. However, a study conducted with a different type of potassium salt, KNO_3_, indicated that the exothermic reaction associated with crystallization was too small to be detected, and only punctual heat release points were recorded (Vazquez et al., 2018). Therefore, we consider it unlikely that the observed decrease in concentration is significantly influenced by heat of crystallization. Further research is needed to fully clarify the underlying mechanisms and resolve which hypothesis best explains this phenomenon. Finally, it should be noted that the salt precipitation on the injection surface did not terminate the evaporation, therefore dry-out continued through the rest of the sample after these time steps.
• The decrease in the growth rate of the salt precipitation on the surface likely coincides with the point when capillary-driven backflow could no longer supply brine to the injection surface. This is likely caused by a loss of connectivity in the evaporating brine films. When the precipitation of salt on the injection surface stops, salt precipitates initially in the pores near the injection surface (Fig. 2-d) and then in the pores away from the injection surface at the subsequent time point of injection (Fig. 2-e). Here, we also observed that micropores acted as a reservoir for brine, and the remaining brine in micropores is transported to the grain-pore boundaries and therefore contributes to precipitation (pink dashed circles *t* = 6.6 h to 9 h in Fig. 3). Such interactions between micro and macropores are well established for heterogeneous sandstones (Ott et al., 2014; Akindipe et al., 2021). Our results show that this effect can play an important role even for very homogeneous sandstones.
• The gas injection continued until no significant changes in the subsequent images were observed. During this final stage of the experiment, salt precipitation on the injection surface maintained its volume, as its rate of change approached zero after 9 h of injection until the end of the experiment (Fig. 4-a).

Due to the main observations on the evolution of the dry-out process having similar trends, corresponding results of samples 2 and 3 were given in the Supplementary Materials (Figs. S3–S5 for sample 2, and Figs. S6–S8 for sample 3). With a slight deviation from the results of sample 1, the rate of change of salt saturation on the surface was lower than the rate of change of N_2_ saturation in the pores near the injection surface (Fig. S5-a). This can be explained by the lower salt concentration of brine in sample 2. For sample 3, the overall trend of observations regarding changes in brine concentration and rate of change in N_2_ saturation closely aligns with the findings for sample 1. It is worth noting that the peaks of the rate of change of N_2_ saturation near the injection surface do not fully align with those of the salt saturation at the injection surface (Fig. S8-a). This discrepancy may be attributed to precipitation in the pressure transducer line (i.e. an experimental artifact), which brings uncertainty into the comparison.

### 3.2 Effect of initial brine concentration on the dry-out

To investigate the influence of brine concentration on the precipitation dynamics, KI solutions with concentrations of 10 and 20 wt. % were used for samples 1, and 2, respectively. A constant N_2_ injection flow rate of 0.8 ml/min was maintained for both sets of experiments, representing the high flow rate case in this study.

**Fig. 5:**
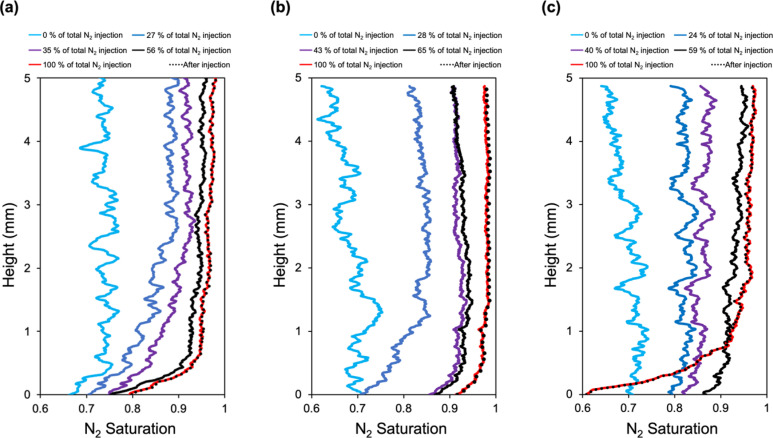
Spatiotemporal change in the N_2_ saturation for (a) sample 1, with a high flow rate and high initial salt concentration, (b) sample 2, with a high flow rate and low initial salt concentration, (c) sample 3, with low flow rate and high initial salt concentration.

Complete dry-out of samples was recorded after 4800 and 4000 PVs of gas injection for the high and low salt concentration cases, respectively. In the following, the amount of gas injected for each time step was normalized by the total volume injected throughout the experiment. The spatial distribution of N_2_ saturation after injection termination is indicated by dashed points in Fig. 5. Overlapping profiles of the after-precipitation and last injection steps in each case confirm a complete dry-out, as shown in Fig. 5 (a-c). The initial time step after drainage corresponds to 73 % and 68 % average N_2_ saturation for high and low-concentration cases, respectively.

Spatial distributions of N_2_ in the pore space at different time points for high (Fig. 5-a) and low (Fig. 5-b) salt concentrations suggest that the evolution of dry-out was similar in both cases. Saturation profiles (before dry-out was reached) show uniform distribution over the length, validating the existence of capillary backflow. During the initial time steps, the gradient in the saturation between the upper and bottom regions of the sample can be linked to the drying front; however, due to the complex interactions that occurred between subflorescence and efflorescence (Section 3.1), the scale of the drying front diminishes at the end of the experiment.

Regarding the effect of salt concentration on the drying rate: during the first two injection steps, the change in overall saturation was slightly lower in the high-concentration case compared to the low-concentration case. This aligns well with expectations, as higher salinity reduces the equilibrium vapor pressure between the gas and liquid phases, resulting in a decreased evaporation rate (Shokri-Kuehni et al., 2017a). However, the time needed to reach complete dry-out is increased afterward, as a low-concentration salt solution has more water to evaporate to attain the critical solubility limit. Hence, the high salt concentration, which shortens the time required to reach the solubility limit, eventually induced the first signs of precipitation more rapidly than the low-concentration case: it occurred when 56 % of total gas injection was reached (*t* = 8.9 h), whereas a similar trend happened at 65 % (*t* = 11.5 h) for the low concentration case. Nevertheless, the total drying time for samples 1 and 2 did not differ significantly; the high-concentration (sample 1) and low-concentration (sample 2) cases required 16 and 17 h, respectively, to reach complete dry-out. This suggests that while brine salinity influenced the dynamics of salt precipitation, it did not notably affect the total drying times under the same flow rate.

Reducing the salt concentration by half yielded a nearly half decrease in the precipitated salt volume. The final data points corresponding to the post-injection in Fig. 5-a and 5-b indicate that 4.31 ∓ 1.09 % (*n* = 5) and 2.81 ∓ 0.88 % (*n* = 5) of the pore spaces are occupied by the precipitated salt, respectively. The change in the concentration did not significantly affect the location where salt precipitates in the sample. Most of the salt precipitated near the injection surface in both cases.

### 3.3 Effect of injection flow rate

Fig. 5-c shows the spatial N_2_ distribution when the injection flow rate was decreased to 0.08 ml/min while brine concentration was kept at 20 wt.%. Until the sample reaches dry out in high and low flow rate cases, 4800 and 3500 PVs of N_2_ were injected, respectively. Initial N_2_ saturation at the beginning of the dry-out for sample 3 was 69 %.

Lower injection rates reduce the pressure gradient effect caused by viscous forces and enhance the effect of capillary flow. Additionally, lower injection rates correspond to a lower rate of evaporation, providing more time for capillary backflow and diffusion to occur. The effect of capillary-driven backflow is observed to be sustained on a longer time scale for the low flow rate case compared to the high flow rate case: saturation profiles followed a similar flat pattern almost until the end of the complete dry-out (Fig. 5-c). The sharp increase in the salt volume at the end of dry out suggests that salt precipitation is extended beyond the pores that were previously occupied by residual brine to include pores filled by capillary-driven backflow. The effect of capillary backflow in similar precipitation patterns has been previously reported (Peysson et al., 2014) In addition, a low flow rate caused higher salt precipitation than that in the high flow rate case, which is again due to the higher impact of capillary backflow. Precipitated salt occupied 6.9 ∓ 1.6 % (*n* = 5) of the pore space of the sample in the field of view, predominantly in the region close to the injection surface. Lower injection rate increased the amount of precipitated salt in comparison to higher injection rate, where 4.31 ∓ 1.09 % (*n* = 5) of the pore space filled with salt precipitation. This result is consistent with findings from (Akindipe et al., 2021). The drying rate was substantially lower than the higher injection flow rate (sample 1). Reducing the flow rate to 0.08 ml/min increased the time needed for complete dry-out by 87 h. This aligns well with expectations, as increasing the flow rate leads to a higher evaporation rate over a shorter evaporation period (Akindipe et al., 2021; Norouzi et al., 2021).

### 3.4 Precipitated salt distribution in the pore space over the entire sample and its effect on porosity and permeability

**Fig. 6:**
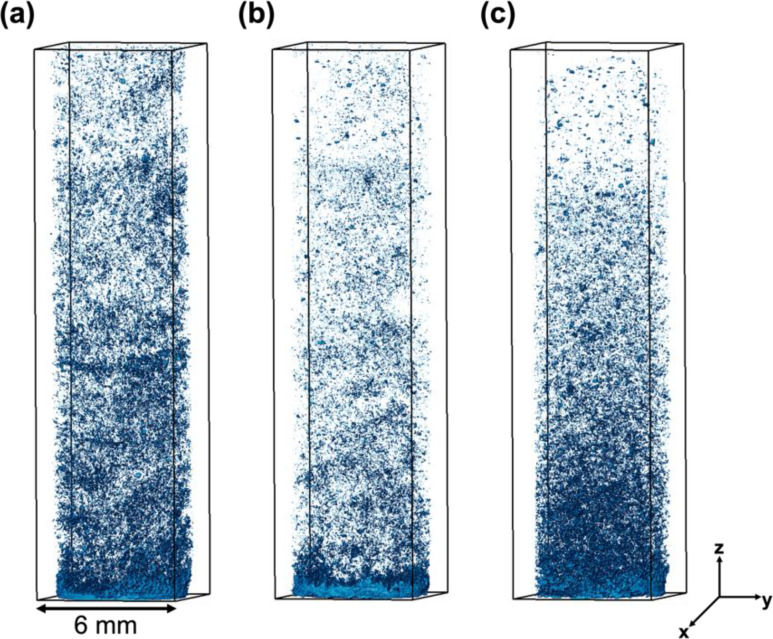
Distribution of salt in the pore space after dry-out. (a) Sample 1, with a high flow rate and high initial salt concentration, (b) sample 2, with a high flow rate and low initial salt concentration, (c) sample 3, with a low flow rate and high initial salt concentration.

For each set, once the bottom part of the sample scanned during the gas injection reached dry-out, images of the full sample were acquired. This was done to verify that the entire sample had reached the final state with no changes in phase saturations related to dry-out. The amount of salt volume precipitated in the entire sample after drying confirms the findings on the subsections imaged at higher resolution. For the same injection rate (Fig. 6-a and b), the higher salinity (Fig. 6-a) caused a higher salt precipitation than the lower salinity (Fig. 6-b) due to having a higher amount of salt in the initial salt solution. When the concentration of the salt solution was kept constant (Fig. 6-a and c), a slightly higher salt volume was observed at a lower flow rate case (Fig. 6-c).

As expected, based on the flow rates utilized, a heterogeneous distribution of salt was observed by the images of after-dry-out states (Fig. 6). In all cases, the volume of salt close to the injection surface is higher than the salt precipitated in the rest of the sample. Nonetheless, the regions away from the injection surface show a slightly different behavior to the change in the injection flow rate. Particularly, in the high flow rate case (Fig. 6-a), salt clusters are visible in regions away from the injection surface, indicating more local precipitation patterns. Given that the samples are extracted from the same region of a Bentheimer sandstone block, the level of heterogeneity, which can induce differences in precipitation patterns, is expected to be similar. The change in the flow rate thus induces slightly different distributions of salt volume in the parts where less precipitation occurs in comparison to the overall distribution.

**Fig. 7:**
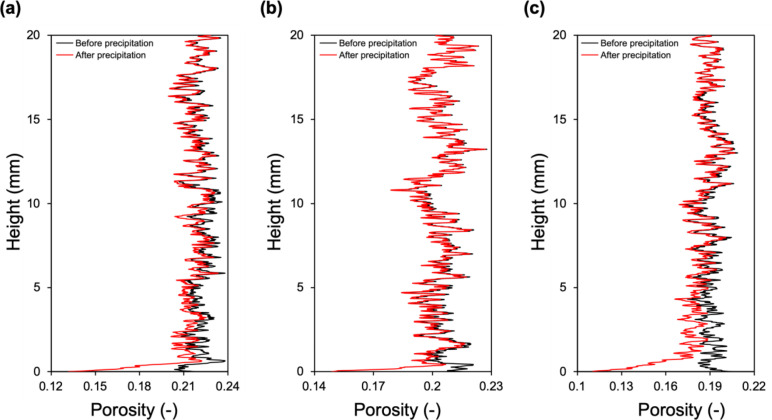
Salt-induced change in macroporosity for the entire length of the sample. (a) Sample 1, with a high flow rate and high initial salt concentration, (b) sample 2, with a high flow rate and low initial salt concentration, (c) sample 3, with a low flow rate and high initial salt concentration.

For a more quantitative interpretation of the salt distribution through the entire pore space, changes in the porosities of the samples at the dry and post-precipitation states were represented in Fig. 7. Due to the very small pore size of the salt crust, which exceeds the resolution capabilities of the micro-CT setup used in the experiments, we are unable to provide details about the pore structure and porosity of the crust. For all samples, salt precipitation caused porosity reductions of less than 3 %, which are lower than the reported values (Tang et al., 2015). One reason could be that high-concentration brine in some pores near the injection surface was possibly attracted by the porous structure of the precipitated salt on the surface as described in Section 3.1, which highlights the effect of efflorescence on the salt precipitation dynamics. Another reason can be the relatively low initial brine saturations of the samples at the start of evaporative drying (Zhang et al., 2020). Low initial brine saturation conditions were purposefully chosen to resemble realistic storage scenarios where cushion gas is maintained in the reservoir to minimize gas saturation changes during storage cycles. Salt precipitation caused more significant porosity reduction close to the injection surface in comparison to the rest of the entire sample. This is expected based on the selected flow rates, which falls in the advective dominated regime. To quantify this reduction, we analyzed a one millimeter region of each sample close to the injection surface. In this region, the high flow rate, high salt concentration case (Fig. 7-a) caused 12 % reduction in porosity. For the same injection flow rate but with half the salt concentration, a 9 % reduction in porosity was observed (Fig. 7-b). When the flow rate is decreased to 0.08 ml/min under high initial salt concentration conditions, 21 % decrease in porosity was observed close to the injection surface. For the case with the high flow rate, overall reductions in the porosity of the entire samples were 1.88 ∓ 0.3 % (*n* = 5), and 0.87 ∓ 0.16 % (*n* = 5) for high (Fig. 7-a) and low salinities (Fig. 7-b), respectively. When the injection rate was decreased with the same initial high salt concentration, porosity was reduced by 2.5 ∓ 0.18 % (*n* = 5) due to the higher amount of salt volume precipitated (Fig. 7-c).

To evaluate the influence of change in porosity on permeability, the N_2_ permeability was measured both in the dry and after-precipitation state. Measurements showed that a 1.88 ∓ 0.3 % decrease in porosity of the entire sample together with the salt crystals on the injection surface caused a 20 % decrease in permeability. As the permeability measurements were conducted on the sample with externally precipitated salt (crust) present in the surface, it is not possible to distinguish the individual contributions of salt precipitation within the pores and on the surface (crust) to the overall permeability reduction. However, the presence of this external layer may have an important role in injectivity reduction, as it creates an additional barrier for the gas flow. By conceptualizing the sample and salt crust as two distinct layers where flow in series occur, we can approximate the minimum permeability of the crust by using Eq. (3). In this equation, *L_sample_* corresponds to the sample length given in Table 1, while *L_crust_* was estimated from the image data to be 0.28 mm (see Supplementary Material Fig. S9 for the dimensions of the crust and sample). Additionally, *K* in Eq. (3) represents the measured permeability after the complete dry-out is reached, which is 1.47 × 10^–12^ m^2^. Assuming that the permeability reduction is entirely caused by the external salt precipitation, the sample permeability after precipitation (*K_sample_*) would be equal to the N_2_ permeability of the sample before salt precipitation, 1.83 × 10^–12^ m^2^. In such case, the crust permeability (*K_crust_*) was estimated to be 7.87 × 10^–14^ m^2^, representing the lower limit of crust permeability. It should be noted that permeability values for the after-precipitation states were not available due to experimental difficulties encountered with the pressure transducer for samples 2 and 3.

(3) $$\frac{L_{crust+sample}}{K}=\;\frac{L_{crust}}{K_{crust}}+\;\frac{L_{sample}}{K_{sample}}$$

## 4 Conclusion

In this study, we conducted dry-out experiments via *in-situ* micro-CT using N_2_ gas in brine-saturated Bentheimer sandstones, mimicking shallow reservoir conditions. Two different flow rates in the advective regime were selected and tested with two different salinities to examine the effect of efflorescence on the dry-out and precipitation dynamics in the pores.

The experiments lead to the following conclusions:

• The tested injection flow rates in the advective regime (Pe>1) consistently resulted in salt precipitation on the surface, regardless of the salinity. Salt accumulation within the pore space was heterogeneous, with higher salt volume observed near the injection surface.
• Capillary backflow had a significant effect in all cases. Re-equilibration of the saturation gradient by capillary backflow resulted in flattened saturation profiles within the pore space. This effect was more pronounced at lower injection rates compared to the higher ones. Observed trend aligns well with the existing literature (Peysson et al., 2014). Once the effect of capillary-driven back flow diminished, the growth rate of salt precipitation on the surface slowed down and precipitation within the pores gradually progressed starting from the pores near the injection surface and extending to those far from the injection surface.
• Salt precipitation occurred in pores containing brine after primary drainage at high injection rates, while at low rates, it extended into the pores which were already invaded by gas flow, indicating a stronger effect of capillary backflow, consistent with previous findings (Norouzi et al., 2021; Bacci et al., 2011).
• At the later stages of drying, we observed an unintuitive behavior in brine concentration, which to our knowledge has not been observed before. The greyscale intensity of brine in the pores, which is directly associated with concentration, significantly reduced. This happened simultaneously with the increase in the growth rate of precipitated salt volume on the surface. Hence, our results suggest a strong correlation in time between these two incidents. Given that the brine becomes undersaturated, it is hypothesized to be driven by the locally high precipitation rates, which is faster than diffusive mixing.
• Our results highlight the role of microporosity even in a very homogeneous sandstone. At the beginning of injection, brine concentration increased more in micropores than in macropores. Salt precipitation was observed in the boundaries of macro and micropores, as microporosity serves as brine reservoirs, which ultimately affect the amount of salt precipitation within the pore space. Such contribution of micropores to salt precipitation is expected to be more pronounced for more heterogeneous rock types.
• Injection flow rate had a decisive role in the time required for complete dry-out. Both high and low salinity cases at the same injection rate reached complete dry-out in similar time scales (∼16 h). A tenfold decrease in the injection rate at high salinity case, however, resulted in almost 6-fold increase in the time required to reach complete dry-out.
• The precipitation-related decrease of porosity in the pore space of the entire sample was less than 3 % for all cases. The reduction in porosities was found to be lower than the existing reported values on porosity reduction (Tang et al., 2015). One possible reason can be the low initial brine saturation in the samples at the start of evaporative drying . Another reason is linked to the porous structure of salt crystals on the surface, which causes highly concentrated brine in macropores to move up to the injection surface. This suggests that efflorescence may influence internal dry-out dynamics and affect the porosity decrease.
• Doubling the salinity resulted in almost a twofold increase in total salt volume, thus a corresponding reduction in porosity, with a minor impact on overall drying dynamics within the tested concentration range. However, the effect of salinity on porosity reduction was less pronounced in the 1-millimeter region near the injection surface. Higher and lower salinities caused 12 % and 9 % reductions in porosity near the injection surface for the same flow rate, respectively. On the other hand, when the flow rate was reduced by a factor of ten while keeping the salinity on the higher end, the decrease in porosity nearly doubled compared to the higher flow rate case. While our findings provide valuable insights into the dynamics under different salinity and flow rate conditions, further work is needed to more reliably extend these conclusions to a wider range of salinities.
• Permeability after dry-out was only measured for the high-concentration, high flow-rate case, revealing a 20 % decrease in permeability. Moreover, the permeability of only the crust itself was estimated to be 7.87 × 10^–14^ m^2^, which is two order of magnitude smaller than the measured gas permeability. How the precipitated salt volume in the pores and on the surface individually contributes to this reduction needs further examination. Additionally, for future research, it is important to clarify the change of relative permeability during dry-out. To this end, a technically more advanced setup that is capable of capturing minor pressure drops across the sample should be designed.

This study reveals that there is a close link between efflorescence and the physics of salt precipitation in the pores in gas injection cases. Salt precipitated on the surface may be more manageable than salt precipitated in the pores for real-field applications; for instance, applying pre-wash treatments can mitigate the risk of complete blockage. To understand the significance of these findings better, further studies are required, particularly involving cyclic gas injection scenarios at different reservoir pressure and temperature conditions, where elevated temperatures affect the rate of salt precipitation by increasing the solubility of water in the gas phase, which accelerates the evaporation process. While we expect similar dynamics governing salt precipitation, the differences in thermophysical properties – mainly viscosity, density, solubility, and interfacial tension – between N_2_ and the gases used in underground storage operations (i.e., CO_2_, H_2_, and CH_4_) are likely to influence drying rate and the extent of salt precipitation.

## CRediT authorship contribution statement

**Gülce Kalyoncu Pakkaner:** Writing – original draft, Visualization, Methodology, Investigation, Formal analysis, Conceptualization. **Veerle Cnudde:** Writing – review & editing, Supervision, Project administration, Funding acquisition, Conceptualization. **Hannelore Derluyn:** Writing – review & editing, Supervision, Project administration, Investigation, Funding acquisition, Conceptualization. **Tom Bultreys:** Writing – review & editing, Writing – original draft, Supervision, Project administration, Methodology, Investigation, Funding acquisition, Conceptualization.

## Declaration of competing interest

The authors declare that they have no known competing financial interests or personal relationships that could have appeared to influence the work reported in this paper.

## Data Availability

Data will be made available on request.

## Appendix Supplementary materials

Image, application 1
