# Supplementary material for: Impact of efflorescence on internal salt precipitation dynamics during injection of gases in porous rocks
Source: Adv Water Resour. 2025 Jul;201:104984. doi: 10.1016/j.advwatres.2025.104984 (PMC12107515; doi:10.1016/j.advwatres.2025.104984)
Supplement: Supplementary file 1 [file mmc1.pdf]

Supplementary Materials

Impact of efflorescence on salt precipitation dynamics during injection of gases in porous rocks

Gülce Kalyoncu Pakkaner[1,2], Veerle Cnudde[1,2,3], Hannelore Derluyn[4], Tom Bultreys[1,2]

[1] PProGRess, Department of Geology, Ghent University, Ghent, Belgium

[2] Centre for X-ray Tomography (UGCT), Ghent University, Ghent, Belgium

[3] Department of Earth Sciences, Utrecht University, Utrecht, The Netherlands

[4] Universite de Pau et des Pays de l 'Adour, E2S UPPA, CNRS, LFCR, Pau, France

1. Bentheimer samples used in experiments

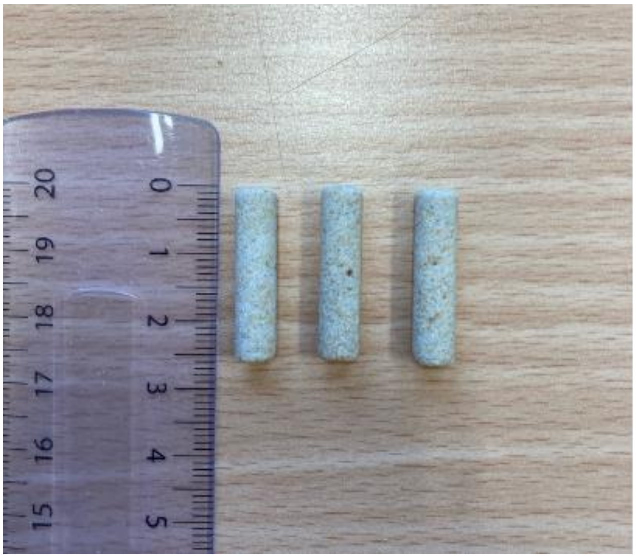

Figure S1. Three Bentheimer samples were drilled from the same block and used in experimental cases 1, 2, and 3.

2. Differential pressure measurements

The end of the primary drainage step was determined using the differential pressure measurements. The ranges of measurements indicating the end of primary drainage for each sample are given in Figure S1. The end of the primary drainage (t = 20 min in Figure S1-a, b, and c) is taken as the initial point for the interpretation of temporal changes in the results during N_2 injection.

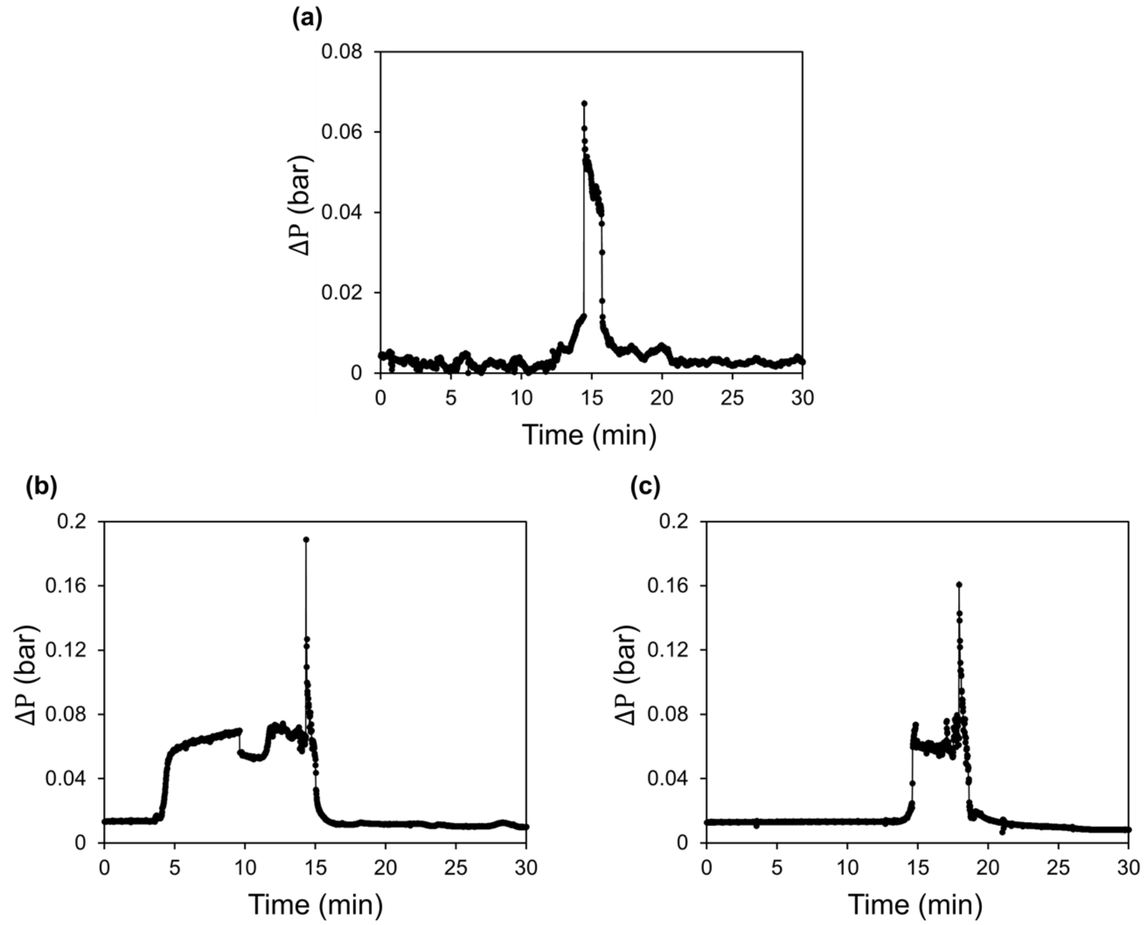

Figure S2. Differential pressure data over time for the duration of primary drainage for experiments conducted with: (a) sample 1: saturated with a 20 wt.% KI solution, injecting N_2 at a constant flow rate of 0.8 after the primary drainage step; (b) sample 2: saturated with a 10 wt.% KI solution, injecting N_2 at a constant flow rate of 0.8 after the primary drainage step; (c) sample 3: saturated with a 20 wt.% KI solution, injecting N_2 at a constant flow rate of 0.08 after the primary drainage step.

3. Observations on the physical mechanisms of salt precipitation for sample 2

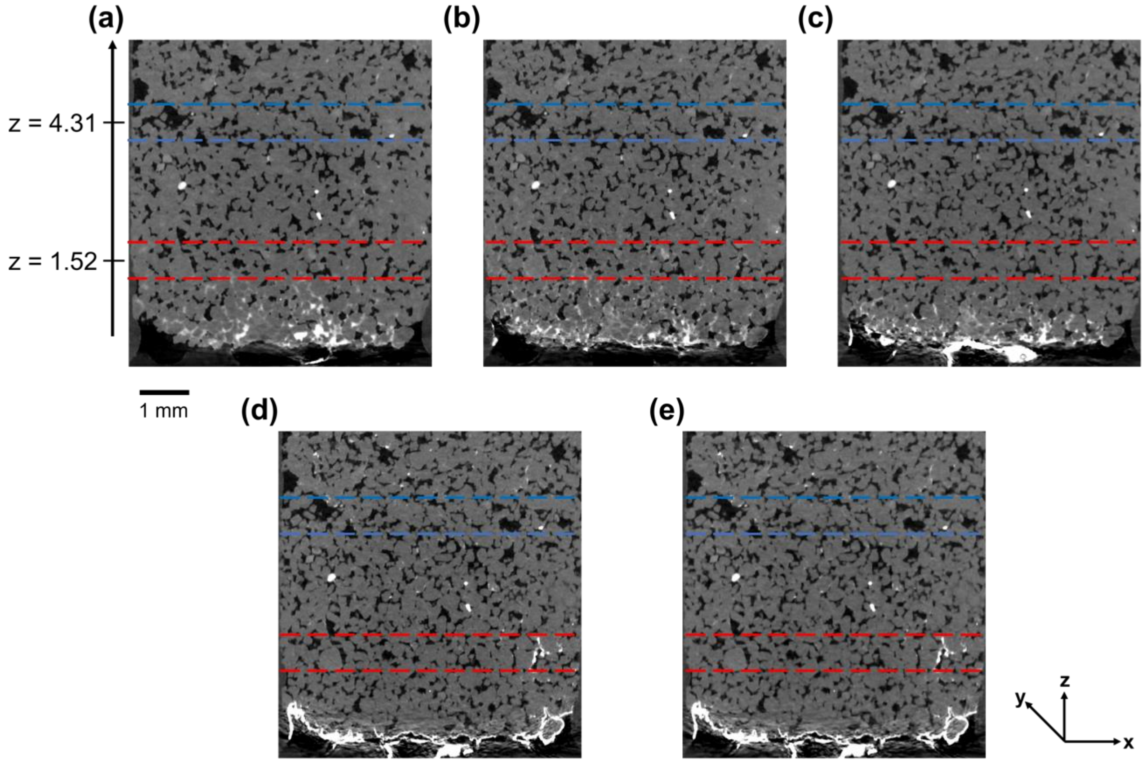

Figure S3. Evolution of the dry-out for sample 2, where N_2 was injected at 0.8 ml/min to the sample initially saturated with a 10 wt.% of KI solution. Bands with blue and red dashed lines represent regions away from and near the injection surface, respectively. z = 0 corresponds to the injection surface. Vertical slices illustrate changes at the following time steps: (a) t =3 h, (b) t = 5 h, (c) t = 5.5 h, (d) t = 7.5 h, and (e) t = 11.5 h.

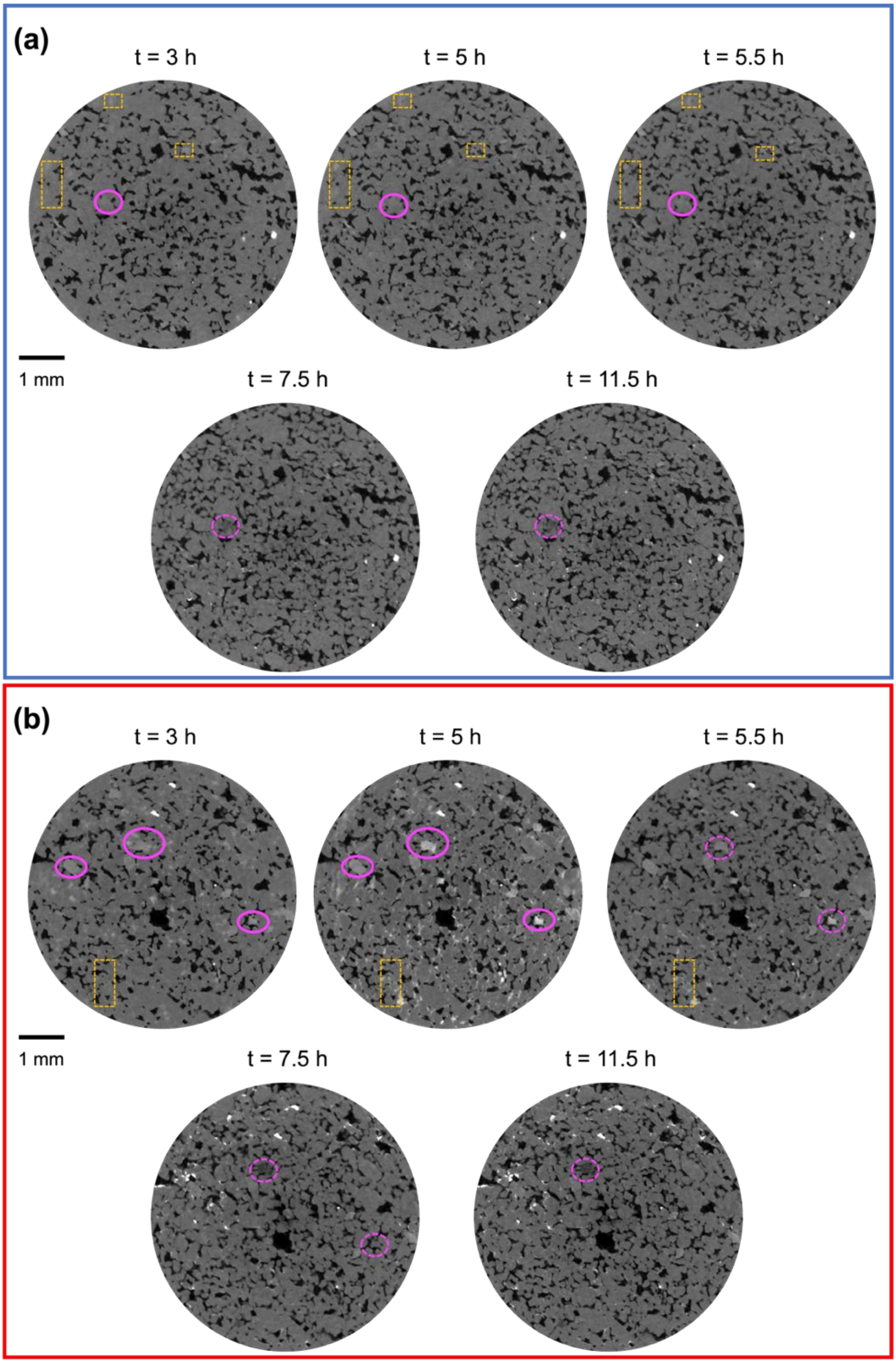

Figure S4. Horizontal slices show the temporal evolution of saturation levels along with the change in the greyscale value of brine. Slices correspond to the position of (a) z = 4.31 mm, and (b) z = 1.52 mm as the middle of blue and red bands given in Figure S2, respectively. Orange dashed boxes and pink dashed and solid circles are provided to follow the explanations given in the main text.

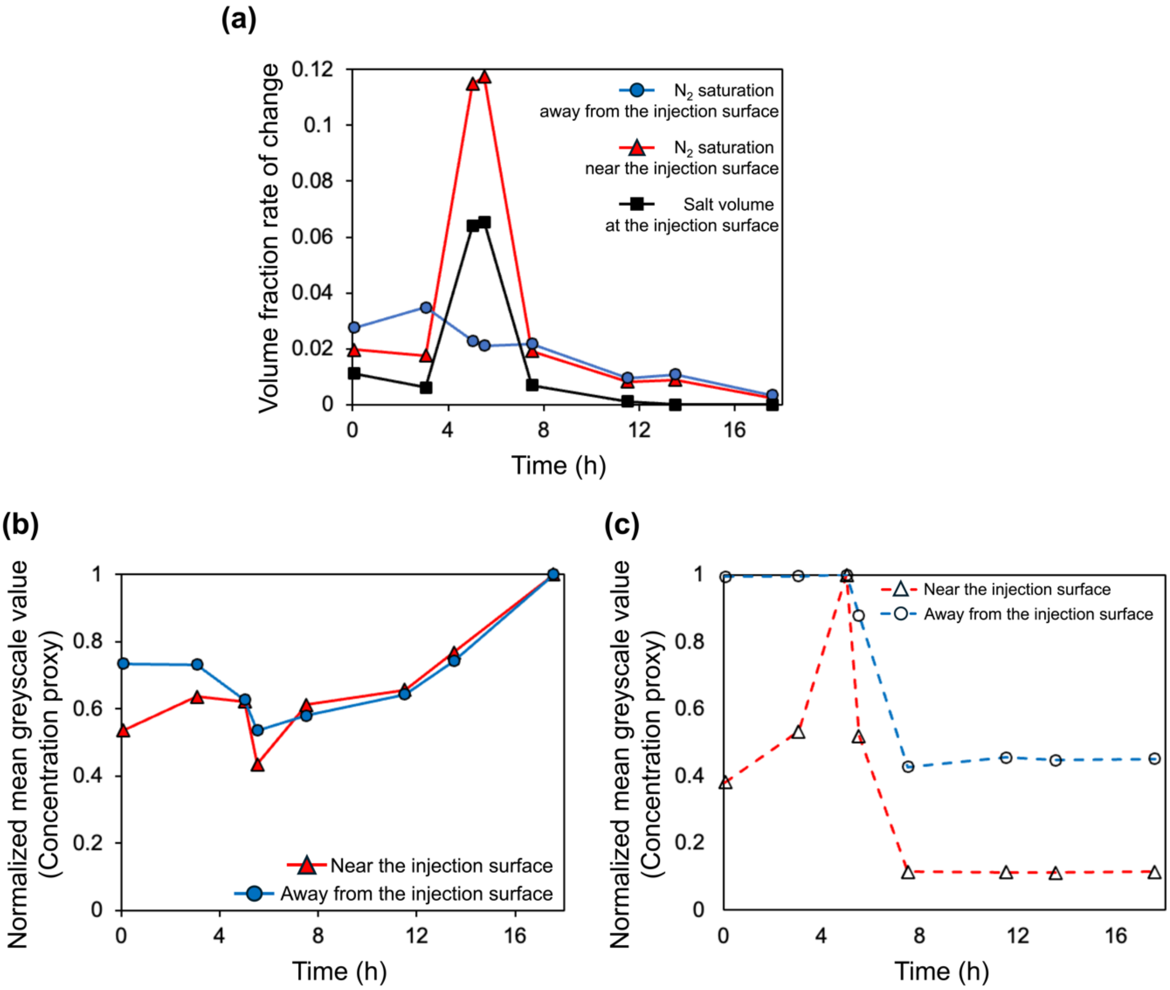

Figure S5. Quantitative interpretation of the summarized dynamics. (a) Rate of change of the average N_2 saturation, together with the rate of change of the volume fraction of the salt crystallization on the surface. Normalized mean greyscale values of high-intensity phase as the concentration proxy for (b) macropores, and (c) micropores in the clay. Blue and red colors represent the average values calculated for the blue and red bands depicted in Figure S2, respectively. The black color in (a) represents the salt crystals on the injection surface.

4. Observations on the physical mechanisms of salt precipitation for sample 3

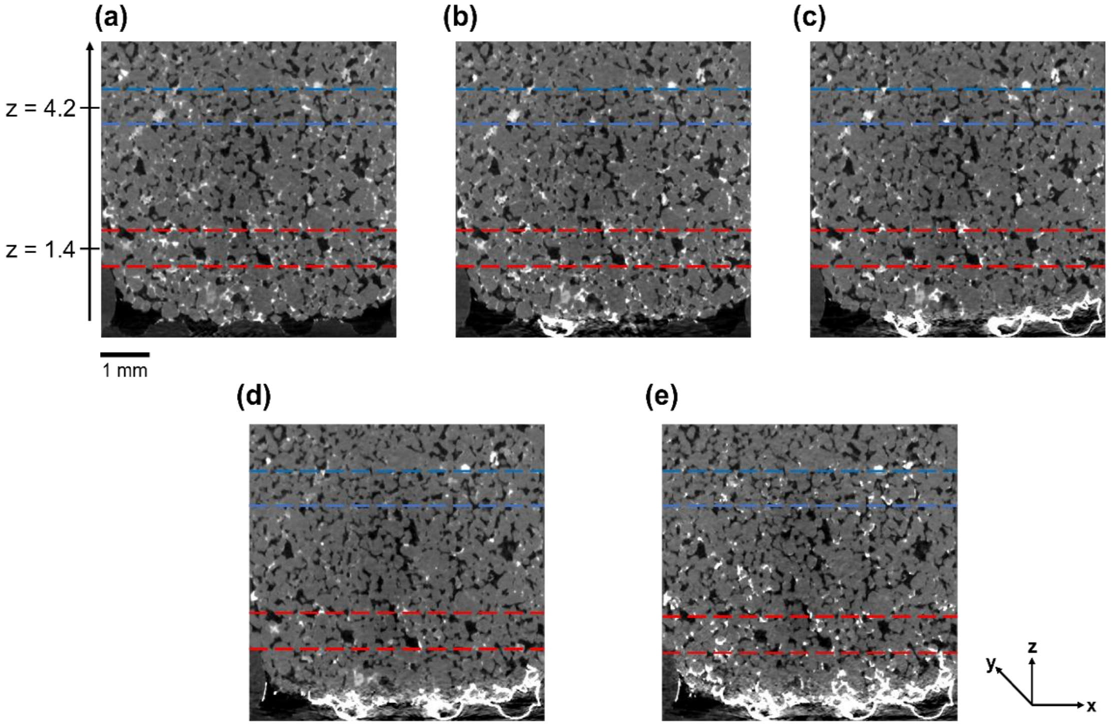

Figure S6. Evolution of the dry-out for sample 3, where N_2 was injected at 0.08 ml/min to the sample initially saturated with a 20 wt.% of KI solution. Bands with blue and red dashed lines represent regions away from and near the injection surface, respectively. z = 0 corresponds to the injection surface. Vertical slices illustrate changes at the following time steps: (a) t =41.6 h, (b) t = 52 h, (c) t = 62 h, (d) t = 68 h, and (e) t = 80 h.

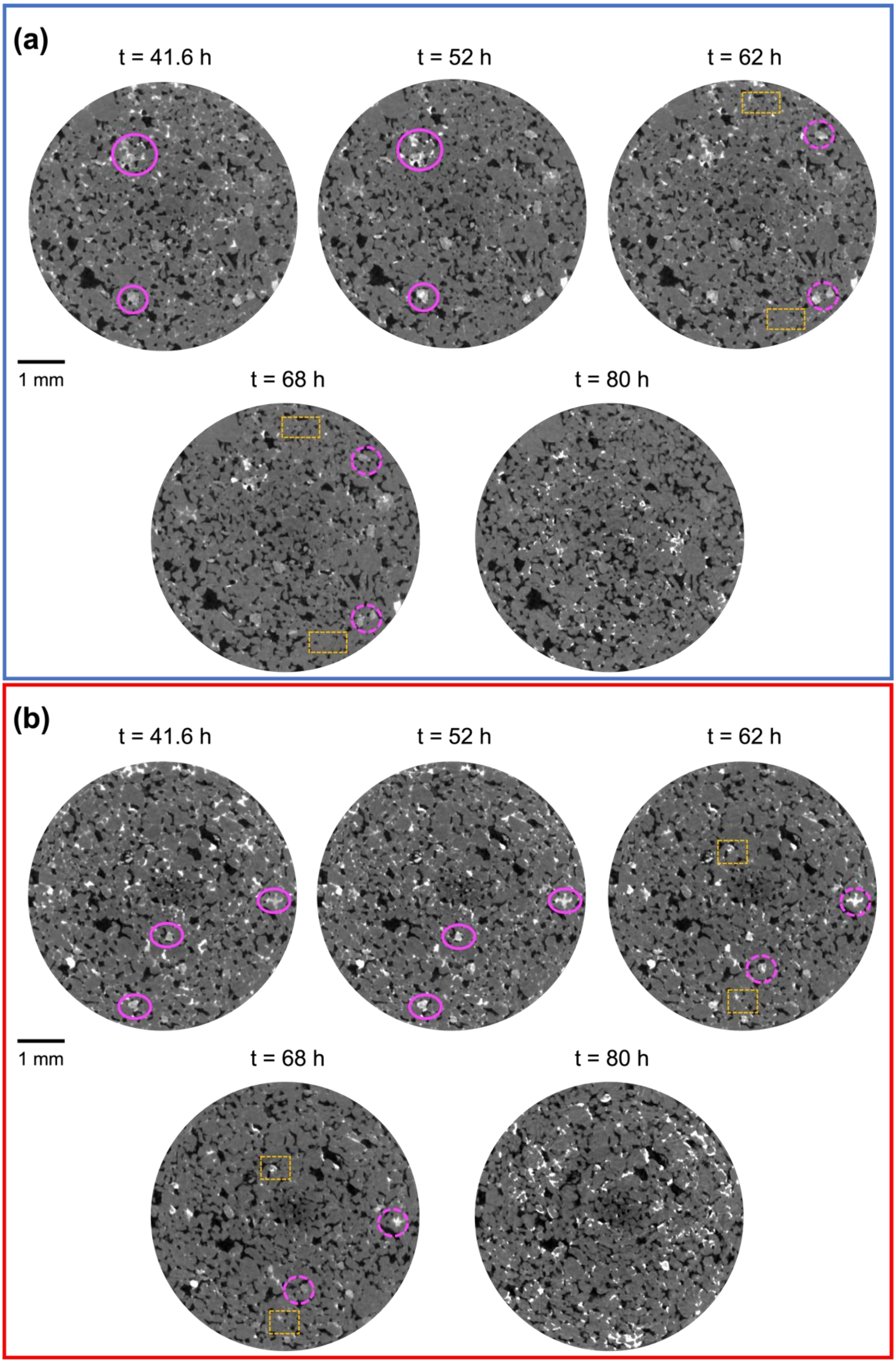

Figure S7. Horizontal slices show the temporal evolution of saturation levels along with the change in the greyscale value of brine. Slices correspond to the position of (a) z = 4.2 mm, and (b) z = 1.4 mm as the middle of blue and red bands given in Figure S5, respectively.

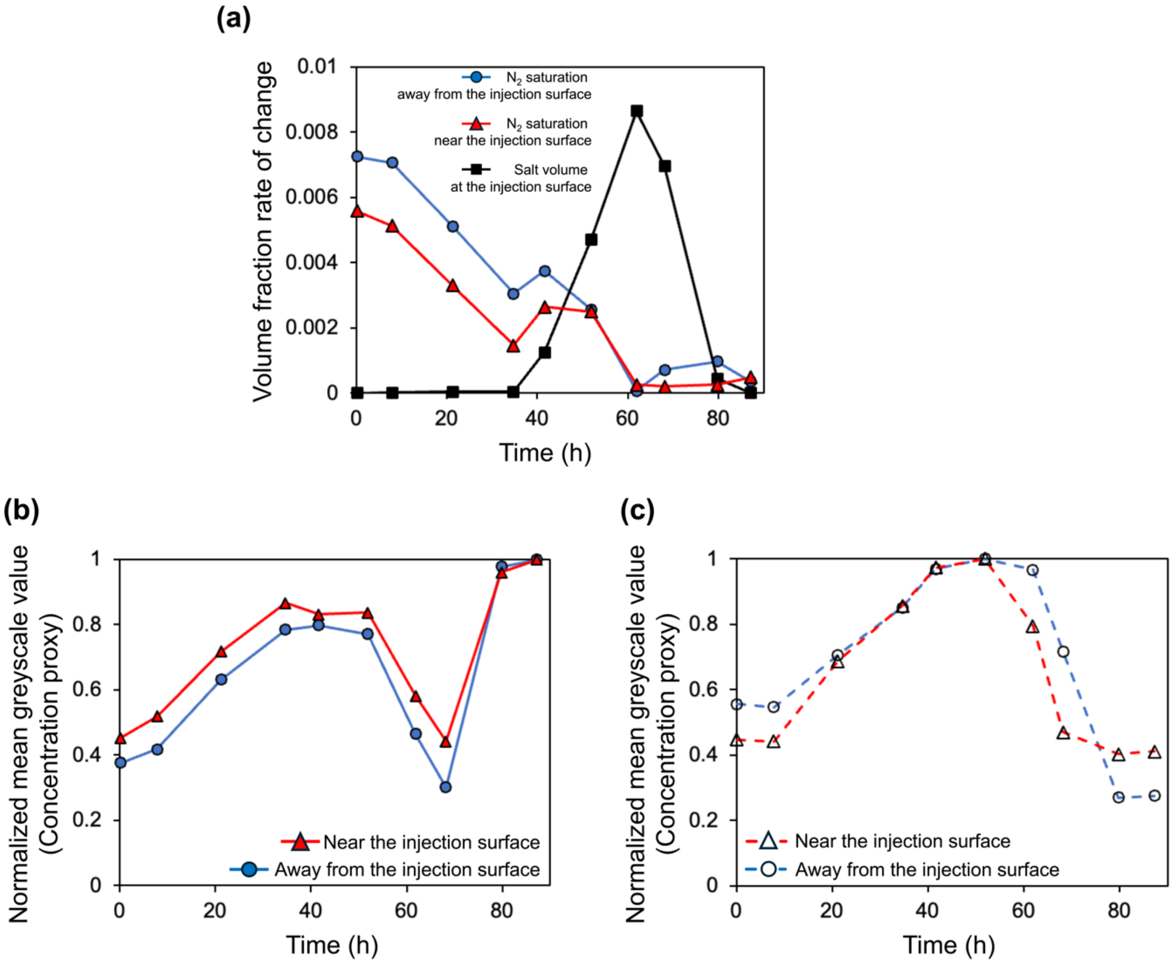

Figure S8. Quantitative interpretation of the summarized dynamics. (a) Rate of change of the average N_2 saturation, together with the rate of change of the volume fraction of the salt crystallization on the surface. Normalized mean greyscale values of high-intensity phase as the concentration proxy for (b) macropores, and (c) micropores in the clay. Blue and red colors represent the average values calculated for the blue and red bands depicted in Figure S5, respectively. The black color in (a) represents the salt crystals on the injection surface.

5. Greyscale image representing the sample and crust length

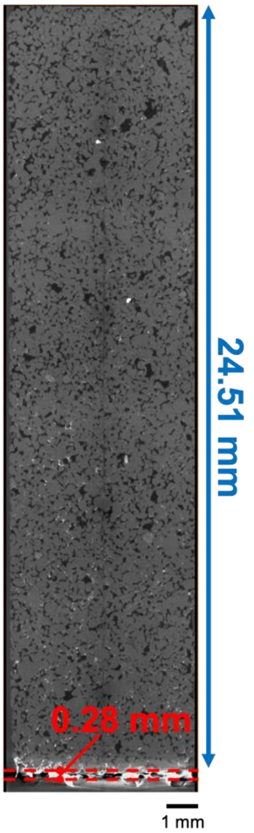

Figure S9. The individual lengths of the sample (given in blue) and the crust (given in red) for Sample 1.

6. Sensitivity analyses on segmentation

Sensitivity analyses for the segmentation of the high-intensity phase were conducted to compute the standard deviations in the salt saturation and proxy concentration at the injection surface and macropores, respectively. Standard deviation calculations were made for a single time point selected for each sample and are given in Table S1.

Standard deviation in the proxy concentrations was computed as follows: mean greyscale intensity values were obtained through over- and under-segmentation of the high-intensity phase. Image data at the time in which brine has the highest greyscale intensity value across all time points was segmented using the same intensity range. Then, the standard deviation of proxy concentration values that were computed for different intensity threshold values was calculated.

Similarly, the standard deviation in the volume fraction rate of change of salt saturation at the injection surface was calculated for the time point where the rate of change of salt saturation reaches its peak. Within the specified range of intensity threshold, the salt phase was segmented at the previous and next time steps surrounding the selected time point, and the rate of change of salt saturation was calculated.

Table S1. Standard deviation values for the variables for different samples.

Variable	Sample 1	Sample 2	Sample 3
Intensity range for segmentation (-)	25000-27000	24600-26400	25200-27200
Timepoint selected for proxy concentration (h)	15	13.5	80
Proxy concentration at the macropores near the injection surface (-)	0.99 ∓ 0.0002	0.76 ∓ 0.0007	0.95 ∓ 0.0002
Proxy concentration at the macropores away from the injection surface (-)	0.99 ∓ 0.0003	0.74∓ 0.0009	0.95 ∓ 0.0004
Timepoint selected for salt saturation on the surface (h)	4.3	5.5	62
Rate of salt saturation (-)	0.05 ∓ 0.007	0.07 ∓ 0.007	0.01 ∓ 0.002
